# Supplementary material for: The genome sequence of a jewel beetle, Agrilus biguttatus (Fabricius, 1776)
Source: Wellcome Open Res. 2024 Jul 26;9:413. [Version 1] doi: 10.12688/wellcomeopenres.22762.1 (PMC11474228; doi:10.12688/wellcomeopenres.22762.1)
Supplement: Peer review history

# Reviewer report: Reviewer response for version 1

**DOI:** 10.21956/wellcomeopenres.25066.r95568

**Published:** 2024-10-16

**Authors:** Themistoklis Giannoulis

**Affiliations:** 1 University of Thessaly, Larissa, Greece

The authors have clearly presented the goal, the methods and the results of their research. Their work is part of a bigger project which is a very impressive effort of "1 species-1 genome". My only question to the authors is the possibility of functionally annotating the genome in a future work.

Are sufficient details of methods and materials provided to allow replication by others?

Yes

Is the rationale for creating the dataset(s) clearly described?

Yes

Are the datasets clearly presented in a useable and accessible format?

Yes

Are the protocols appropriate and is the work technically sound?

Yes

Reviewer Expertise:

Genetics, animal genomics, heat stress, dairy cows

I confirm that I have read this submission and believe that I have an appropriate level of expertise to confirm that it is of an acceptable scientific standard.

# Reviewer report: Reviewer response for version 1

**DOI:** 10.21956/wellcomeopenres.25066.r103790

**Published:** 2024-10-15

**Authors:** Nikoletta Andrea Nagy

**Affiliations:** 1 University of Debrecen, Debrecen, Hungary

The manuscript is well written and the genome presented is of high quality and can be a useful contribution to future research on the spread and adaptation of Agrilus biguttatus to different conditions.

**Minor comment:**

 Background

 change: "It is a very distinctive bright" -> "It has a very distinctive bright"

Are sufficient details of methods and materials provided to allow replication by others?

Yes

Is the rationale for creating the dataset(s) clearly described?

Yes

Are the datasets clearly presented in a useable and accessible format?

Yes

Are the protocols appropriate and is the work technically sound?

Yes

Reviewer Expertise:

Insect genomics

I confirm that I have read this submission and believe that I have an appropriate level of expertise to confirm that it is of an acceptable scientific standard.

# Reviewer report: Reviewer response for version 1

**DOI:** 10.21956/wellcomeopenres.25066.r103791

**Published:** 2024-10-14

**Authors:** Bhagya c. Thimmappa

**Affiliations:** 1 USDA Agricultural Research Service (Ringgold ID: 17123), Chatsworth, New Jersey, USA

In the work "The genome sequence of a jewel beetle, *Agrilus biguttatus *(Fabricius, 1776)," the authors report the nuclear and mitochondrial genome assembly for female *Agrilus biguttatus*, an oak-boring beetle. The reported nuclear genome assembly spans 368.10 MB and is nearly complete, with a BUSCO value of 97.8%. The reported mitochondrial genome assembly is 17.41 kb in length.

 The manuscript is well written, with enough introduction and a clear method section. The reported nuclear genome assembly is a great resource for understanding the effects of habitats, diets, and temperature on these beetles. Furthermore, the mitochondrial genome could aid in evolutionary studies. The authors also mentioned that the nuclear genome assembly will be annotated, a great resource for omics and other analyses.

**Minor comment:**

1. Like nuclear genome assembly annotation, mitochondrial genome annotation could be a great resource for evolutionary studies.

Are sufficient details of methods and materials provided to allow replication by others?

Yes

Is the rationale for creating the dataset(s) clearly described?

Yes

Are the datasets clearly presented in a useable and accessible format?

Yes

Are the protocols appropriate and is the work technically sound?

Yes

Reviewer Expertise:

Genomics, Metagenomics, Transcriptomics, Bioinformatics, Plant-Microbe Interactions.

I confirm that I have read this submission and believe that I have an appropriate level of expertise to confirm that it is of an acceptable scientific standard.

# Reviewer report: Reviewer response for version 1

**DOI:** 10.21956/wellcomeopenres.25066.r93699

**Published:** 2024-08-26

**Authors:** Komal Kumar Bollepogu Raja

**Affiliations:** 1 Department of Pathology & Immunology, Baylor College of Medicine, Houston, TX, USA

The manuscript by Garland et al., presents a high-quality genome assembly of the jewel beetle *Agrilus biguttatus* *.* This work is a significant contribution to the genomic resources available for this species. The authors used both Pacific Biosciences HiFi long reads and Hi-C data, which ensures comprehensive and accurate assembly. The study shows an impressive N50 of 34.5 megabases and chromosomal-level scaffolding. The integration of the mitochondrial genome as a single contig further enhances the assembly’s completeness. The quality metrics presented in the report, including a BUSCO score of 97.8% and a k-mer completeness of 100.0%, demonstrate the robustness and thoroughness of the assembly process. The detailed methodology and manual curation contribute to the high reliability of the data, which will be valuable for future studies on *A. biguttatus* and related species.

 Overall, this genome assembly represents a substantial advance in our understanding of *A. biguttatus* and will be an important resource for researchers investigating its genetics, ecology, and evolutionary biology. The manuscript is well-prepared with good English and grammar. It offers a solid foundation for subsequent research in beetles.

 I have a minor comment. Discussing in few sentences on how this genome data could impact or integrate with broader research contexts or comparative studies with other beetle genomes will benefit the manuscript.

Are sufficient details of methods and materials provided to allow replication by others?

Yes

Is the rationale for creating the dataset(s) clearly described?

Yes

Are the datasets clearly presented in a useable and accessible format?

Yes

Are the protocols appropriate and is the work technically sound?

Yes

Reviewer Expertise:

Genomics, Bioinformatics, Single cell omics and Developmental biology

I confirm that I have read this submission and believe that I have an appropriate level of expertise to confirm that it is of an acceptable scientific standard.
